# Patterning of Organic Semiconductors Leads to Functional Integration: From Unit Device to Integrated Electronics

**DOI:** 10.3390/polym16182613

**Published:** 2024-09-15

**Authors:** Wangmyung Choi, Yeo Eun Kim, Hocheon Yoo

**Affiliations:** 1 Department of Semiconductor Engineering, Gachon University, Seongnam 13120, Republic of Korea; 2 Department of Electronic Engineering, Gachon University, Seongnam 13120, Republic of Korea

**Keywords:** organic semiconductor patterning, organic semiconductor crystallinity, surface-grafting polymer, capillary force lithography, wettability, evaporation assistant, diffusion

## Abstract

The use of organic semiconductors in electronic devices, including transistors, sensors, and memories, unlocks innovative possibilities such as streamlined fabrication processes, enhanced mechanical flexibility, and potential new applications. Nevertheless, the increasing technical demand for patterning organic semiconductors requires greater integration and functional implementation. This paper overviews recent efforts to pattern organic semiconductors compatible with electronic devices. The review categorizes the contributions of organic semiconductor patterning approaches, such as surface-grafting polymers, capillary force lithography, wettability, evaporation, and diffusion in organic semiconductor-based transistors and sensors, offering a timely perspective on unconventional approaches to enable the patterning of organic semiconductors with a strong focus on the advantages of organic semiconductor utilization. In addition, this review explores the opportunities and challenges of organic semiconductor-based integration, emphasizing the issues related to patterning and interconnection.

## 1. Introduction

Organic semiconductors (OSCs) combine the electronic properties of semiconductors and organic compounds [1,2,3,4,5]. Efforts regarding OSCs are actively being made because of their flexibility and even stretchable properties [6,7,8,9]. Furthermore, they provide a low-cost process and easy control of physical properties using thin film deposition techniques [10,11,12,13], making them highly advantageous for various applications. OSCs are used in a wide range of devices, including light-emitting diodes [14,15,16], photodetectors [17,18,19,20], and thin film transistors [21,22,23,24]. In addition to the conventional functions mentioned above, emerging functional devices made from OSCs are currently being investigated, and representative examples include neuromorphic devices [25,26,27,28], physically unclonable functions [29,30,31,32,33], logic devices [34,35,36], and multi-functional sensors [37,38,39,40]. The cost-effective and room-temperature compatibility of solution-based processes can reduce the cost of existing electronic products significantly and enable disposable [41,42,43,44] large-area electronic applications [44,45,46,47]. OSCs typically exist as thin films in devices. Hence, researchers have developed various thin film preparation techniques based on solution processes or vacuum deposition. On the other hand, although commercially successful silicon-based semiconductors are used in industry, OSCs still face integration challenges. To progress toward the commercialization of organic semiconductors, it is essential to improve their sensitivity to environmental stresses such as chemical solvents [48], heat [49], and UV exposure [50] during the patterning process. This improvement is particularly important for large-area integration, which is essential for developing practical applications that require precise patterning processes for OSCs.

Patterning OSCs is challenging because they are incompatible with photoresist-based photolithography processes. Most OSCs are vulnerable to physical and chemical damage from solvents, making it difficult to use conventional photoresists and achieve compatibility with etching and other existing patterning processes. Accordingly, considerable efforts have been made to develop alternative methods for patterning OSCs. Techniques that consider the vulnerability of OSCs by avoiding conventional photoresist and etching processes have been developed. Patterning OSCs is being reported by controlling the chemical and physical energy between organic semiconductor materials and substrates [51,52,53,54]. This patterning technique not only avoids the need for photoresist and etching processes but also improves the crystallinity and crystal orientation of the organic semiconductor [55,56,57,58,59,60], reducing defects and enhancing charge transport [61,62,63,64,65]. These advances have significantly contributed to the development of OSCs from the unit device to the integration of multiple devices, extending their application to fields where the physical properties of organic materials change due to external energy, such as in-time temperature integrator devices [66,67,68] and fluorescence sensors [69,70,71].

In this context, this paper presents a focused review of various approaches to pattern OSCs, emphasizing the control of the chemical and physical energy between organic semiconductor materials and substrates. This review provides a comprehensive summary of principles underlying the induction of patterns in OSCs, including surface energy control of substrates, chemical reactions of materials, and interactions among solvents. Furthermore, it provides specific examples of controlling crystallinity in OSCs. Furthermore, application examples are provided for electronic components, such as transistors and sensors, utilizing patterned OSCs. This review contributes to the understanding and advancement of patterning techniques critical for integrating OSCs into practical electronic devices.

## 2. Patterning Methods of Organic Materials

### 2.1. Surface-Grafting Polymers

**Figure 1:**
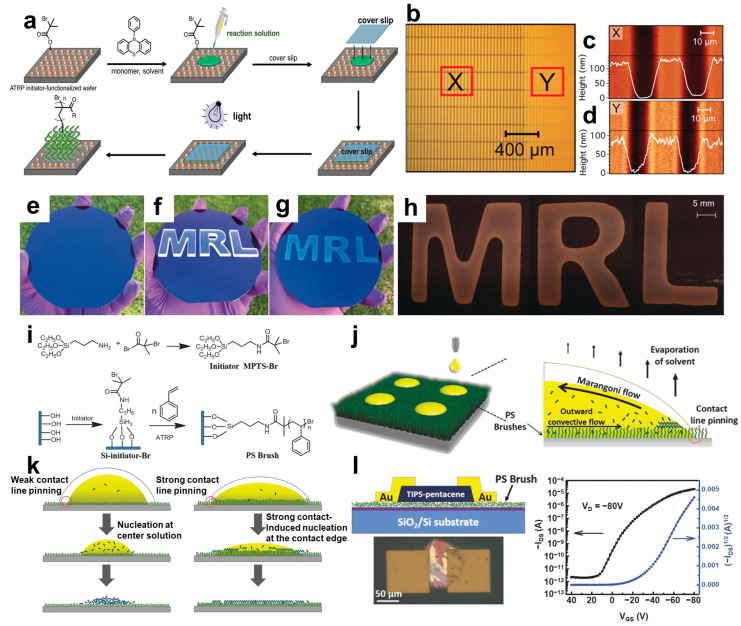
(**a**) Schematic diagram of visible light-mediated p(DMAEMA) brush growth under ambient conditions. (**b**) Optical image of photomask-guided sequential p(DMAEMA) growth on a wafer-based substrate patterned with underlying polymers (X: substrate, Y: patterned polymer). Atomic force microscopy topography images and height profiles for patterned p(DMAEMA), at (**c**) X region (substrate) and (**d**) Y region (patterned polymer). The p(DMAEMA) brush synthesis process under ambient conditions using M, R, and L letter glass covers: (**e**) initial chemically treated silicon wafer, (**f**) polymer brush growth process through M, R, and L letter covers, (**g**) p(DMAEMA) brushes patterned with the letters M, R, and L. (**h**) Enlarged optical image of p(DMAEMA) brushes patterned in the shape of MRL [54]. Copyright © 2018, John Wiley and Sons. (**i**) Schematic diagram of the PS-brush synthesis process. (**j**) Schematic diagram of the evaporation process for organic semiconductor crystal growth between the PS brushes and organic semiconductor ink. (**k**) Mechanism of organic semiconductor crystal growth depending on the chain length of PS brushes. (**l**) Schematic diagram of the device and transfer curve of the TIPS-pentacene-based TFT with optimized crystallinity [57]. Copyright © 2016, John Wiley and Sons.

Surface-grafting polymers are produced using a technique that induces covalent bonding between a substrate and an organic material through chemical treatment, allowing the organic material to grow [72,73,74,75]. This method enables localized chemical treatments, facilitating the patterning of organic materials [76]. The methodologies for producing surface-grafting polymers can be classified into “grafting to” and “grafting from” methods. “Grafting to” methods involve the terminal group of a preformed polymer binding to a chemically modified substrate surface, resulting in the polymer chain grafting onto the substrate surface [77,78]. “Grafting from” methods involve polymer chains being grafted via surface-initiated polymerization reactions of monomers with an initiator chemically bound to the substrate surface [79,80]. This approach allows simple surface energy control, which is useful for patterning through partial chemical reactions. Narupai et al. reported a strategy for applying a precursor solution containing a mixture of a monomer and photoredox catalyst onto a surface-initiated silicon wafer to pattern the polymer brushes of 2-(dimethylamino)ethyl methacrylate (DMAEMA), followed by covering with a glass cover and exposure to light (Figure 1a) [54]. The initiator was undecyl-2-bromo-2-methylpropanoate, with a visible LED with a 405 nm wavelength applied to activate the photocatalyst. The glass cover acts as a barrier to oxygen, facilitating polymer growth in an inert atmosphere within a glovebox or a chamber designed for specific environments. This strategy allows the straightforward patterning of organic materials without expensive equipment. Intricate organic patterns with a resolution of 20 × 200 μm can be achieved by extending the glass cover to serve as a photomask (Figure 1b). Interestingly, p(DMAEMA) patterned on a 20 × 200 μm scale exhibited uniform polymer patterning regardless of the presence of a patterned polymer in the underlying layer (X: substrate, Y: patterned polymer), as shown in Figure 1c,d. Under ambient conditions, large-scale p(DMAEMA) patterning was achieved on a 4-inch silicon wafer functionalized with the initiator by simultaneously using coverslips with the shapes of the letters ‘M’, ‘R’, and ‘L’ (Figure 1e–h). Following the proposed strategy, 200 μL of a monomer precursor solution was dropped, and individual letter cover slips were placed. Under 405 nm LED illumination, polymer patterns in the shapes of the letters were formed, demonstrating spatial control over the patterning process. Based on these results, various iridium-based monomers that emit under 254 nm illumination were grafted simultaneously, producing multicolored patterned polymer brushes.

The surface-grafting polymer method can improve the crystal quality and morphology of OSCs by controlling the chain length of the grafted polymers. Improvements in the crystallinity of inkjet-printed small organic semiconductor films and their influence on device performance by controlling the chain length of grafted polystyrene (PS) was reported by Ge et al. [57]. Figure 1i shows the method for synthesizing PS brushes, where the PS chain lengths were variably synthesized by controlling polymerization times at 100 °C. PS grafting onto SiO_2_/Si substrates enhances compatibility with the organic solvent tetralin, resulting in strong pinning of the three-phase contact line (Figure 1j). Between the PS brush and organic semiconductor ink, strong pinning of the three-phase contact line and heterogeneous nucleation enhance the crystallization of the organic semiconductor by restricting the outward migration of organic molecules. As a result, organic molecules crystallize from the edges toward the center, preventing the “coffee ring” effect during crystallization. Based on this mechanism, Figure 1k presents a schematic diagram of the crystal growth of OSCs as a function of the PS brush chain length. As the grafted polymer chain length becomes longer, the surface energy of the PS brush increases, causing the printed ink to spread over a wider diameter. This phenomenon enhances the crystallization rate at the receding contact line as the solvent evaporates. Using a PS brush with a chain length of 5.14 mm, 6,13-Bis(triisopropylsilylethynyl)pentacene (TIPS-pentacene) was printed to achieve a mobility of 0.35 ± 0.23 cm^2^·V^−1^·S^−1^, demonstrating the fabrication of organic thin film transistors (Figure 1l).

### 2.2. Capillary Force Lithography

**Figure 2:**
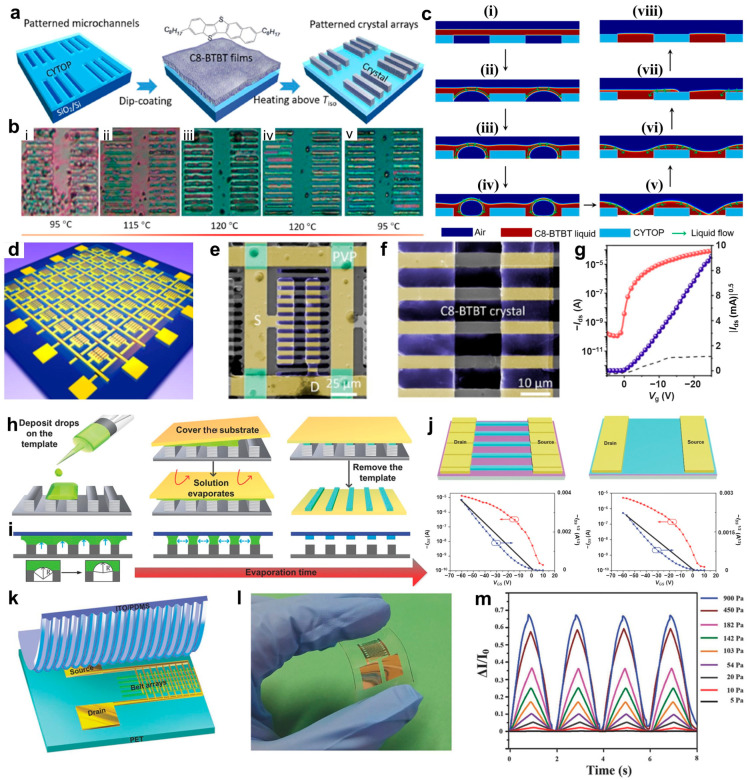
Capillary force-based (**a**) schematic diagram of the C8-BTBT patterning process, (**b**) sequential optical microscopy images of the formation process of C8-BTBT crystals (i: solid; ii and iii: phase transition; iv: microchannel formation; v: cooling), and (**c**) COMSOL multiphysics simulation of liquid C8-BTBT molecule flow (i: C8-BTBT deposition; ii: C8-BTBT liquid moves along the sidewall of CYTOP; iii: C8-BTBT liquid starts to fill the bottoms; iv and v: as the C8-BTBT liquid fills the droplets form an upward concave meniscus; vi and vii: C8-BTBT liquid fills the voids due to capillary forces; viii: C8-BTBT microchannel formation). (**d**) Schematic diagram of a 13 × 13 array of OTFTs based on single-crystal C8-BTBT. At the unit device scale, (**e**,**f**) scanning electron microscopy images of a single-crystal C8-BTBT-based OFET and (**g**) transfer curve (red line: logarithmic scale; purple line: linear scale; dashed line: leakage current) [55]. Copyright © 2020, Elsevier. 1D single-crystal C8-BTBT array synthesis: (**h**) schematic diagram and (**i**) mechanism. (**j**) Transfer curves of OFETs based on single-crystal C8-BTBT morphology (left OFET: 1D single-crystalline belt arrays; right OFET: thin film; red line: logarithmic scale; blue line: linear scale). 1D single-crystal C8-BTBT-based pressure sensor: (**k**) schematic diagram, (**l**) photograph of the device, and (**m**) pressure–current response curves [60]. Copyright © 2018, John Wiley and Sons.

Capillary force lithography has been studied extensively to achieve patterning and high-quality crystal formation in OSCs [81,82,83,84]. Capillary force lithography is a method for patterning materials based on the phenomenon occurring where a liquid contacts and ascends along the walls of capillary tubes. The procedure involves depositing a polymer with a glass transition temperature (*T*_g_) onto a substrate, followed by applying an elastomeric mold, such as polydimethylsiloxane (PDMS), on top. As the polymer on the substrate reaches temperatures above its *T*_g_, it undergoes a change in physical properties, forming an isotropic liquid that can spontaneously spread over the wetted surfaces. This results in the filling of voids in the mold due to capillary forces, causing localized patterning [85]. Deng et al. reported high-resolution patterned single-crystal organic TFT arrays based on capillary platforms fabricated by photolithography [55]. Figure 2a presents a schematic diagram of the capillary force-based patterning process for 2,7-dioctyl [1]benzothieno[3,2-b][1]benzothiophene (C8-BTBT). The hydrophobic CYTOP mold, fabricated using etching and photolithography techniques, revealed localized hydrophilic SiO_2_ surfaces, inducing a significant wetting contrast that drives the patterning of organic materials. C8-BTBT was dispersed in dichloromethane and deposited onto the substrate in film form as a deep coating. The film was heated above the *T*_g_ of C8-BTBT (*T* = 120 °C). At this temperature, the C8-BTBT film transitions from a solid to a liquid state, allowing it to flow into the voids of the CYTOP mold and form a patterned 1D crystal array (Figure 2b). Patterning was achieved in a structure where capillary forces opposed gravity, demonstrating the dominance of capillary forces in the process. Figure 2c shows the mechanism of the proposed process. The voids of the CYTOP mold are filled with air (Figure 2c(i)). Upon heating above its *T*_g_, the liquefied C8-BTBT flows downward along the hydrophilic plasma-treated CYTOP sidewalls (Figure 2c(ii)). As the liquid C8-BTBT flows along the sidewalls, it wets the hydrophilic SiO_2_ surface and forms droplets (Figure 2c(iii)). These droplets transform into upward concave meniscus shapes as the SiO_2_ surface is gradually filled with liquid C8-BTBT (Figure 2c(iv,v)). The liquid C8-BTBT on CYTOP moves into the voids of the mold because of capillary forces (Figure 2c(vi,vii)), forming a uniformly distributed C8-BTBT liquid over time (Figure 2c(viii)). OSCs patterned using capillary force lithography were fabricated into organic thin film transistors (OTFTs) arranged in a 13 × 13 array (Figure 2d). The single-crystal C8-BTBT in the unit device exhibited a well-organized form without defects such as voids (Figure 2e,f). The fabricated TFTs achieved a mobility of 5.78 cm^2^·V^−1^·S^−1^ and an on/off ratio of ~10^6^ (Figure 2g).

One of the techniques widely used in organic semiconductor patterning is lithographically controlled wetting (LCW), which utilizes capillary forces driven by surface tension in the formed capillaries [86,87,88]. In 2018, Zhao et al. developed organic field-effect transistors (OFETs) by producing one-dimensional crystalline C8-BTBT patterns using capillary bridges fabricated through photolithography and ion etching [60]. Capillary forces were induced through the LCW technique using micropillar-structure bridges (capillary bridges) with controlled wettability differences between the top and sidewalls, allowing the organic semiconductor solution to form unidirectionally aligned nanowires at the tops of the capillary bridges. The sidewalls of the fabricated hydrophobic template were modified to make them hydrophobic using heptadecafluorodecyltrimethoxysilane (FAS) molecules. The organic semiconductor was patterned by dropping a precursor solution onto the asymmetrically wettable topographical capillary bridges, which continuously covered the flat substrate (Figure 2h). The liquid between the pillars and the substrate of the capillary bridges exhibited capillary action with an upward concave meniscus as the solution evaporated (Figure 2i). As the solvent evaporated, the concentration of the liquid increased, with capillary flow from the asymmetrically wettable pillars directed toward the substrate, resulting in the nucleation and growth of the organic semiconductor on the target substrate. A single-crystal C8-BTBT belt array OFET was fabricated using this phenomenon. The performance enhancement of this device was confirmed through a comparison with OFETs based on spin-coated C8-BTBT thin films (Figure 2j). At the same gate voltage (*V*_GS_), the patterned single-crystal C8-BTBT belt array OFET achieved a higher drain current (*I*_DS_) than the thin film C8-BTBT OFET, with the hole mobility increasing more than 79-fold. The performance-validated C8-BTBT belt array was transferred onto a PET substrate for application as a pressure sensor (Figure 2k,l). The C8-BTBT belt array used as a flexible channel exhibited stable current variations when pressure was applied to the device (Figure 2m).

### 2.3. Wettability

**Figure 3:**
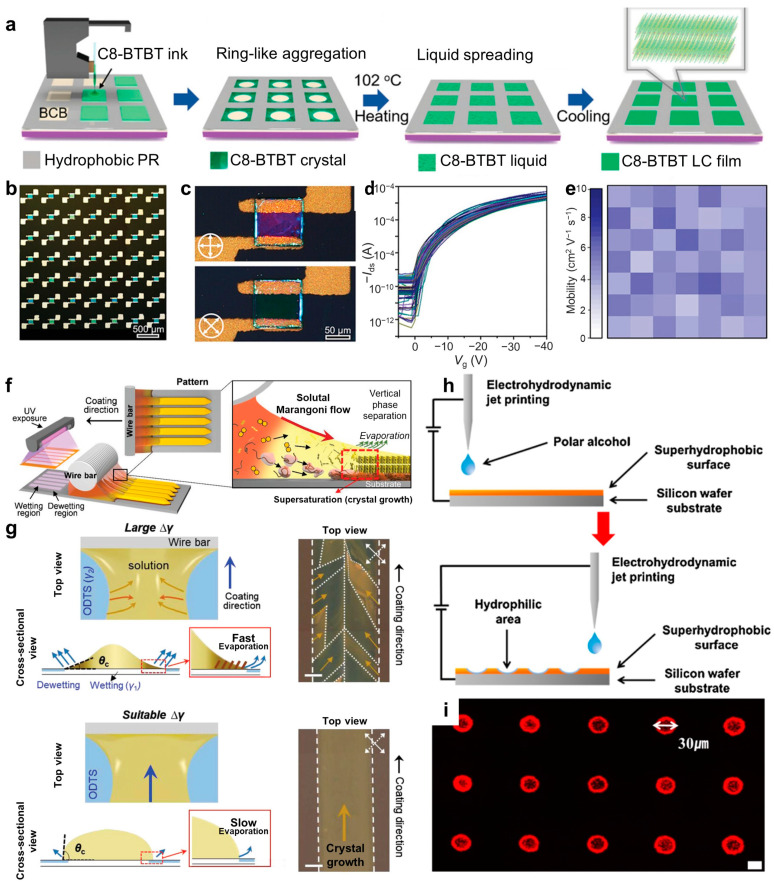
(**a**) Schematic diagram showing the patterning process for C8-BTBT LC films using the IJP-MP method. (**b**) Polarized optical microscopy image of a 7 × 7 OFET array patterned with C8-BTBT LC film. (**c**) Polarized optical microscopy images of a typical OFET device. (**d**) Transfer characteristics measured from 49 OFETs within the 7 × 7 device array. (**e**) Spatial distribution of saturation mobilities across the 49 OFET devices [51]. Copyright © 2021, John Wiley and Sons. (**f**) Schematic diagram showing the fabrication of patterned OSC crystals on a wettability-controlled substrate using the bar-coating method. The inset details the pattern design details and an enlarged view of the film crystallization processes. (**g**) Cross-sectional and top views of the meniscus and contact line at the interface between the wetting (white) and dewetting (light blue) regions with high (Δ*γ* ≥ 31.5 mN·m^−1^) and appropriate (Δ*γ* = 21.1 mN·m^−1^) surface tension differences [58]. Copyright © 2021, John Wiley and Sons. (**h**) Polarized optical microscopy images of coated films on substrates with large and small surface tension differences. Preparation of a superhydrophobic surface on a silicon wafer using atmospheric RF plasma, followed by the formation of a selective hydrophilic area using an EHD jet printing system. (**i**) fluorescent image of polystyrene nanoparticles after deposition (scale bar: 50 μm) [106]. Copyright © 2017, Elsevier.

The development of techniques for local control of surface wettability of substrates has allowed the widespread use of these methods in various patterning fields [89,90,91,92]. Wettability is generally classified into hydrophilicity and hydrophobicity. Hydrophilic surfaces exhibit a high affinity for water, resulting in a contact angle of less than 90°. Hydrophobic surfaces, however, have a low affinity for water and a contact angle of 90° or more, tending to repel water. Through local control of the surface wettability of the substrate through external chemical or physical treatments, the OSC solution can form patterned films with specific shapes as a result of growing along the adjusted surface energy regions. Hydrophilic surfaces are used in thin film coating industries to deposit aqueous liquids uniformly, while hydrophobic surfaces are used to restrict deposition areas and control pattern sizes in inkjet printing industries [93,94,95,96]. The ability to control the wettability of substrates provides a simple and flexible method to modify surfaces into desired patterns, leading to broad application possibilities in the lab-on-a-chip field. Fang et al. presented a method for patterning C8-BTBT liquid crystalline (LC) thin films in high-performance organic integrated circuits based on the synergistic combination of inkjet printing and melting treatment (IJP-MP) [51]. This approach uses wetting/non-wetting patterned surfaces to achieve precise spatial control of the LC solution during inkjet printing, which allows the production of high-quality C8-BTBT LC crystals and high-resolution patterns. On the other hand, in the conventional inkjet printing process, as the solution ejected from the nozzle onto the substrate dries, the solute tends to move toward the edges, causing the coffee-ring effect [97]. This can lead to lower crystal quality and poor crystal orientation [94,97]. Figure 3a presents a schematic diagram of the process of IJP-MP for patterning C8-BTBT LC films. Initially, a C8-BTBT solution ink (≈200 pL) was printed selectively onto a mold constructed from a hydrophobic photoresist. The frameworks within the mold were composed of divinyltetramethyldisiloxane bis(benzocyclobutene) (BCB), which possesses hydrophilic properties. As a result, the deposited ink droplets were confined to the hydrophilic areas and did not spread into the hydrophobic patterned photoresist.

As the ink gradually evaporated, ring-shaped crystalline aggregates formed at the edges of the frameworks due to the well-known “coffee ring” effect. Subsequently, the sample was heated above the melting point of C8-BTBT (*T* = 102 °C) in ambient air, causing the aggregated crystals to transform into an isotropic liquid phase, which spread spontaneously to form a thin liquid film within the framework. The sample was then slowly cooled to 95 °C at a rate of 1 °C min^−1^, allowing a phase transition from the isotropic liquid phase to the crystalline phase. Finally, the C8-BTBT LC films formed within each framework, resulting in a high-resolution patterned structure. Figure 3b presents an optical microscopy image of an OFET array fabricated with patterned C8-BTBT LC films. The patterned C8-BTBT LC film formed a uniform thin film without voids (Figure 3c). Figure 3d plots the overlapping transfer curves of 49 OFETs measured in the saturation region. All devices operated correctly, achieving 100% yield with similar transfer curves, showing that the patterned OSC layer effectively minimized crosstalk between devices. The carrier mobility distribution of the 7 × 7 OFET array had a uniform color range (Figure 3e), confirming the high-quality patterning of C8-BTBT LC films achieved using the IJP-MP method.

In 2021, Lee et al. introduced a novel method for depositing OSC single-crystal thin films using a gap-controlled bar-coating method combined with a wetting patterning strategy and soluble Marangoni flow [58]. This simple bar-coating approach forms arrays of single-crystal OSC thin film patterns at speeds of millimeters per second [58,98,99,100]. Figure 3f presents a schematic diagram of the bar-coating system for processing patterned OSC thin films. The SiO_2_/Si surface was patterned using octadecyltrichlorosilane (ODTS) to induce the selective wetting of a C8-BTBT/poly[bis(4-phenyl)(2,4,6-trimethyl-phenyl)amine] (PTAA)/toluene ink. The patterned areas were exposed to ultraviolet/ozone (UV/O_3_) for an appropriate time. The gap between the substrate and the coating bar was maintained at 10 μm, into which the ink was placed. As the bar moved, the solvent (i.e., toluene mixture) began to evaporate along the meniscus line formed in the bar–substrate gap, and the solutes (C8-BTBT and PTAA) were supersaturated along the solution contact line. Supersaturation increased the nucleation and crystal growth of C8-BTBT molecules. The active ink contains solvent additives (1 vol%) that can influence the flow at the contact lines because of their different physical properties compared to the primary solvent. The concentration of additives can vary according to the location, resulting in a surface tension gradient in the solution. This surface tension gradient, induced by the mixed solvent, generates Marangoni flow along the meniscus line [101,102,103]. This solutal Marangoni flow can improve film uniformity and promote the formation of a high-crystallinity patterned OSC film formation by enhancing vertical phase separation [104,105]. 

A significant difference in surface energy between the patterned and non-patterned regions of the substrate is detrimental to crystal growth. The solution does not adhere selectively to the printed patterns when the surface energy difference between the wetting and dewetting regions is small (Δ*γ* ≤ 21.1 mN m^−1^) because of the weak differences in wettability. In contrast, a large surface energy difference between the wetting and dewetting regions (Δ*γ* ≥ 31.5 mN·m^−1^) results in additional nucleation along the pattern walls, yielding polycrystalline C8-BTBT growth (Figure 3g). This occurs because the solution spreads from the dewetting region to the wetting region, forming a small contact angle (*θ*_c_ ≤ 10°). The small *θ*_c_ indicates rapid solvent evaporation and supersaturation at the pattern edges, leading to additional (undesirable) nucleation sites and randomly oriented polycrystalline growth. With an appropriate difference in surface energy (Δ*γ* = 21.1 mN·m^−1^), the solution can still maintain a high *θ*_c_ at the pattern edges. These results extend the understanding of the fundamental fluid dynamics and crystallization processes of OSC molecules in the gap-controlled bar-coating system.

As another example, a simple and efficient selective surface modification method using atmospheric pressure RF plasma (apRFp) and electrohydrodynamic (EHD) jet printing was reported by Lee et al. [106]. apRFp can discharge at atmospheric pressure and room temperature, allowing a simple and cost-effective modification process. This method allows easy control of surface wettability by selecting appropriate gases and processing conditions [107,108,109]. The EHD jet printing technology overcomes limitations by controlling the electric field between the nozzle and substrate to emit droplets or jets of various volumes [110,111,112]. Thus, EHD technology can be used to fabricate small patterns ranging from the microscale to the nanoscale using solutions of almost any viscosity.

Figure 3h presents the process of locally patterning the hydrophilicity of a substrate using apRFp and EHD jet printing. Radicals from aldehyde groups generated by apRFp attach to the substrate surface, forming a superhydrophobic layer. EHD jet printing locally prints polar alcohols, such as butyl alcohol, onto substrates with modified hydrophilic surfaces. In this process, radicals from aldehyde groups on the substrate surface chemically react with polar alcohols and are removed, making the surface hydrophilic. Areas with different wettabilities can be formed on the same substrate by selectively patterning hydrophilic regions on the surface. Templates produced using this technique can have poly(3,4-ethylenedioxythiophene)-poly(styrenesulfonate) ink, Luria–Bertani broth containing Salmonella bacteria, or polystyrene fluorescent nanoparticle suspension solutions deposited onto the hydrophilic regions by immersing them in the solutions or applying the solutions dropwise. Polystyrene nanoparticles were coated onto the template, and the emitted fluorescence confirmed that the materials had been coated uniformly onto the hydrophilic regions (Figure 3i). EHD jet printing is a promising technology that can reduce undesirable defects, costs, and complex processing steps associated with conventional photolithography and overcome the resolution limitations of traditional inkjet printing techniques. Considering the excellent versatility of this patterning process and the high quality of patterned films, it has great potential for high-performance organic integrated circuits and OFET arrays.

### 2.4. Evaporation Assistant

**Figure 4:**
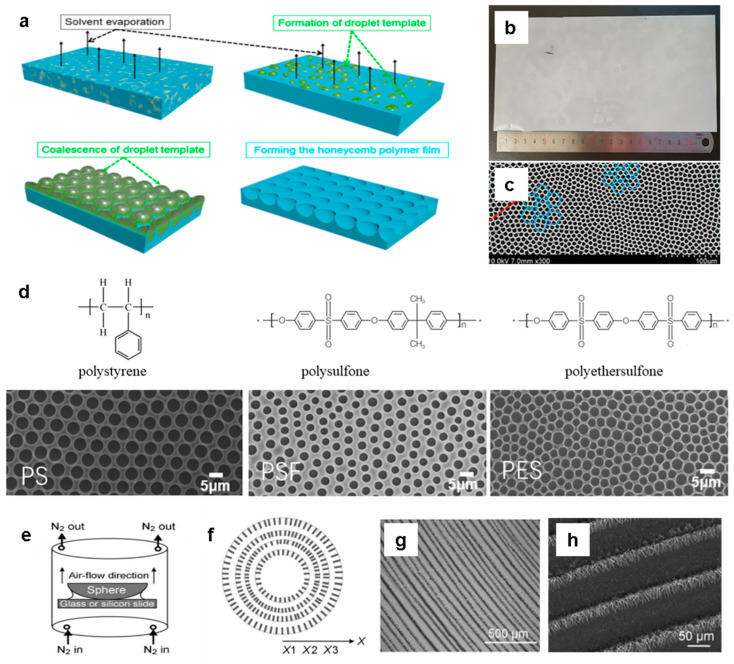
(**a**) Schematic diagram of the formation process of honeycomb-patterned films. (**b**) Large-area honeycomb film for digital photography. (**c**) Scanning electron microscopy image of the honeycomb-patterned film. (**d**) Scanning electron microscopy images of the surfaces of patterned films made from different polymers, along with the molecular structural formulas of these polymers: PS, PSF, and PES [116]. Copyright © 2021, American Chemical Society. (**e**) Illustration of the sphere-on-flat evaporation setup. (**f**) Illustration of the concentric rings of as-prepared organic nanowires. (**g**) Optical micrographs and (**h**) scanning electron microscopy images of concentric rings of DMQA nanowires formed during solvent evaporation [117]. Copyright © 2011, John Wiley and Sons.

Organic semiconductors can be patterned by controlling the flow within evaporating droplets to regulate pattern formation. The evaporation-assisted technique involves mixing a polymer with a solvent that has high solubility for the polymer and a nonsolvent that does not dissolve the polymer. Due to the difference in evaporation rates, the nonsolvent remains, causing the polymer to precipitate and, as the nonsolvent condenses and evaporates, a honeycomb pattern is formed. In addition, they produce concentric ring patterns of organic nanowires by controlling the evaporation of dye solution droplets [113,114,115]. For example, Cheng et al. suggested a large-scale and low-cost method to fabricate ordered films with honeycomb patterns using common commercialized polymers, such as polystyrene (PS), polysulfone (PSF), and poly(ether sulfone) (PES), under normal indoor conditions [116]. As the solvent evaporates, the nonsolvent forms droplet templates on the film surface, resulting in large-scale honeycomb-patterned films. Figure 4a shows the process and mechanism of surface pattern formation. A solvent/nonsolvent mixture was prepared using methylene chloride (solvent) and propylene glycol (nonsolvent) at various ratios, to which a specified amount of polymer was added and dissolved to obtain a casting film solution. Methylene chloride has high volatility, while propylene glycol has low volatility. These characteristics can lead to phase separation in solution. Phase separation occurs as the polymer solution is cast onto the glass substrate and the solvent evaporates, resulting in the nucleation and growth of nonsolvent droplets on the film surface. Due to its surface tension, the nonsolvent propylene glycol forms uniform droplet templates on the polymer surface. As the nonsolvent droplets are removed through continuous drying, a honeycomb pattern emerges on the film surface. This method enabled the large-scale fabrication of honeycomb pattern films (Figure 4b). Figure 4c shows the honeycomb structure across the entire surface of the polymer film in scanning electron microscopy images. Furthermore, pattern size was controlled the using various commercial polymers such as PS, PSF, and PES (Figure 4d). This patterning technique allows the production of honeycomb-shaped films in typical ambient conditions without stabilizers or humidity control. This technique also enables the modification of the surface patterns based on the polymer materials and nonsolvent content. 

As another example, a novel approach to preparing regular concentric rings of aligned organic nanowires by simply evaporating the solvent in constrained geometries was demonstrated by Wang et al. [117]. The density, length, and periodicity of the nanowire arrays can be adjusted by controlling the evaporation rate. Adjusting the initial concentration allows control over the density and spacing of the concentric arrangement of organic nanowires. This easy technique can also fabricate large-scale organic semiconductor devices with nanowire configurations. The evaporation-induced self-assembly (EISA) method relies on the fact that a drop of colloidal solution always leaves ring-like deposits around its perimeter [115,118,119,120,121]. For example, Lin et al. designed a simple method to control droplet evaporation in constrained geometries, leaving well-organized gradient concentric ring patterns [122,123]. Integrating the EISA method with concentric ring patterning achieved the simultaneous self-assembly, alignment, and patterning of organic semiconductor nanowires in a single step. This study revealed the straightforward approach to fabricating large-scale concentric arrays of nanowires by solvent evaporation in constrained geometries. N,N′-dimethylquinacridone (DMQA) was chosen as the nonvolatile solute, and concentric ring patterns of DMQA nanowires were prepared from chloroform solutions at concentrations of 0.2, 0.1, and 0.05 mmol·L^−1^. Approximately 150 μL of a DMQA solution was dropped into the gap between the slides and a lens, with a nitrogen gas stream aiding solvent removal. The evaporation process was conducted at room temperature in a fume hood to control the solvent evaporation direction and rate (Figure 4e). After drying the solution on the substrate, concentric rings with aligned nanowires formed on both surfaces (Figure 4f). Optical microscopy and scanning electron microscopy (Figure 4g,h) revealed the prepared concentric rings at 0.10 mmol·L^−1^, with remarkable regularity over large areas spanning hundreds of micrometers. Each ring consisted of numerous DMQA nanowires. This easy evaporation approach was applied to fabricate well-aligned organic nanowires from homogenous solutions, significantly reducing material consumption and production costs for nanoscale devices. Furthermore, well-organized concentric nanowire rings can form from several other organic compounds, such as 2,4-Bis[4-(N,N-dimethylamino)phenyl] squaraine (SQ) and perylene tetracarboxylic diimide (PTCDI) through evaporation [124]. The self-organized patterns of molecular OSCs over large areas have potential applications in various optoelectronic devices, including OFETs and biosensors.

### 2.5. Diffusion

**Figure 5:**
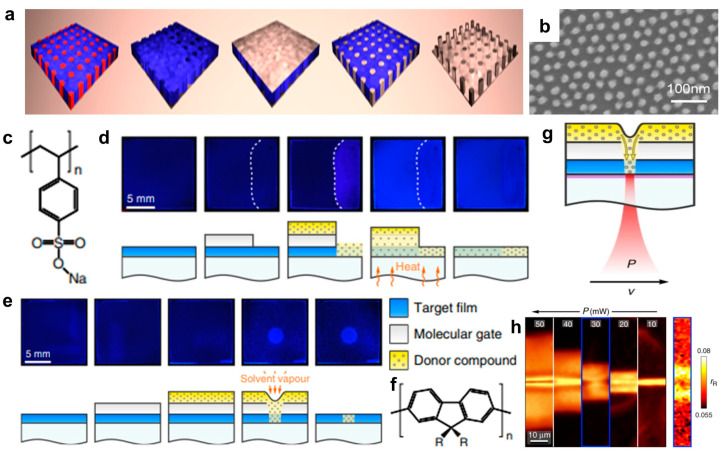
(**a**) Schematic diagram of the preparation procedure for an array of silica pillars. (**b**) Representative scanning electron microscopy image of the patterned silica pillar array on the surface [137]. Copyright © 2015, American Chemical Society. (**c**) Chemical structures of pNaSS. Illustration of the molecular gate concept through PL switching in PFO films, depicted for (**d**) thermal and (**e**) solvent vapor-based stimuli. The top rows show photographs of the films under UV light at various processing steps, with schematic illustrations in the bottom rows. (**f**) Chemical structures of PFO (R = C_8_H_17_). (**g**) Schematic diagram of the laser-patterning process. (**h**) PL intensity at 438 nm showing a spectral selection of β-phase emission. In addition, a Raman intensity ratio image (right panel) of the line patterned using *P* = 30 mW was obtained, indicating the local β-phase fraction as estimated from the ratio of Raman intensities at 1257 and 1606 cm^−1^ [138]. Copyright © 2020, Springer Nature.

Diffusion-based organic semiconductor patterning techniques utilize the selective diffusion properties of organic semiconductor materials in solvents. The diffusion of polymers on solid surfaces has broad implications in various chemical processes, including surface-based biosensors [125,126,127], polymer separations [128], lubrication [129,130,131,132], heterogeneous catalysis [133,134,135], and surface-mediated supramolecular self-assembly [136]. Patterns are formed in organic semiconductors using masks or templates to direct the solvent to specific areas of the organic semiconductor, inducing selective diffusion. The organic semiconductor is patterned only in specific regions by diffusing the solvent along the mask pattern into the organic semiconductor material. High-resolution patterns can be achieved by controlling the diffusion rate and time. This method is suitable for the simple and rapid micro-patterning of organic semiconductors. Wang et al. used polystyrene-b-poly(methyl methacrylate) (PS-b-PMMA) thin films as templates to fabricate an array of silica nanopillars, illustrating the manufacturing process schematically (Figure 5a) [137]. The PS-b-PMMA solution was spin-coated onto a fused-silica surface functionalized with a random PS-r-PMMA copolymer with a PS:PMMA monomer ratio of 64:36. A thin film of block copolymers was self-assembled through thermal annealing, exhibiting PMMA cylindrical microdomains arranged vertically on the surface. Subsequently, the PMMA blocks were removed by UV irradiation and acetic acid washing (nonsolvent for PS but a good solvent for PMMA). The remaining PS template was further treated with oxygen plasma to remove any residual PMMA. A linear PDMS solution was spin-coated onto these PS template films and annealed at 80 °C under vacuum to allow PDMS diffusion into the nanopores. The oxygen plasma treatment decomposed the organic material on the surface, and the PDMS in the nanopores was transformed into silica. Additional etching with CF_4_ plasma was conducted to remove the remaining PDMS. Figure 5b presents representative images of the patterned surface observed by scanning electron microscopy. The columnar pattern surface influenced polymer motion and lateral diffusion in various ways.

For the fine patterning of OSCs, in 2020, Perevedentsev et al. proposed a versatile approach using the local diffusion of functional small molecules through solution-processed “molecular gate” layers [138]. The molecular gate controls the diffusion of functional small molecules into the target film under regulated stimuli. Functional small molecules diffuse using membranes that control permeability, leading to pattern formation. Poly(sodium 4-styrenesulfonate) (pNaSS) was used as the molecular gate material (Figure 5c), with individual processing steps shown in Figure 5d,e. The poly(9,9-dioctylfluorene) (PFO) film spin-coated on a glass substrate exhibits a deep blue PL under UV illumination (Figure 5f). After spin-coating pNaSS in an aqueous solution, a uniform film was formed, with the photoluminescence (PL) characteristics of PFO unchanged. The presence of the pNaSS molecular gate protects the PFO film during the spin-coating of linoleic acid (LA), used as a functional ”solid solvent”. The controlled diffusion of LA is activated by heating it slightly above its melting point (*T*_m_ = 44 °C) or by exposure to solvent vapor. This leads to LA diffusion into the PFO film, locally modifying the PFO chain structure and inducing β-phase formation and subsequent PL changes. Although the glassy PFO emits dark blue fluorescence, the β-phase PFO emitted a brighter blue fluorescence. Exposing the solvent vapor through a nozzle induces localized changes in the chain structure through LA–polymer interactions. The residual LA donor layers and pNaSS gates were removed by spin-off using solvents such as acetone and water. The uniform PL emission color from the sample regions where LA diffused through the gate contrasts with the less uniform color where LA was deposited directly onto PFO, indicating that gate diffusion is a highly controllable process. Laser patterning of the β-phase chains in PFO film was performed in the configuration shown in Figure 5g. Heating an indium tin oxide substrate with a laser controls LA diffusion into the PFO film, with the diffusion being controlled by the position and parameters of the laser. Patterned β-phase lines were produced by scanning the sample with constant writing speed *v* and varying power *P* at successive laser focal planes. PL microscopy images showed that laser power, which controls local temperature rise and consequent functional small molecule diffusion, is a key parameter in patterning features (Figure 5h). Molecular gate-based patterning, maintaining film planarity by donor integration, facilitates direct integration into multilayer structures. It offers the spatial resolution of photolithography and the versatility of printing techniques. Various semiconductor polymers can be spatially patterned by selecting appropriate donor compounds, chain structure and orientation, and material composition.

In this review, we revisited various OSC patterning techniques through the control of chemical and physical energy between the substrate and organic semiconductor materials. Table 1 provides a detailed summary of the OSC patterning techniques in terms of patterning methods, organic semiconductor materials, pattern resolution, and electronic device types and performance parameters.

## 3. Conclusions and Outlook

The development of advanced patterning techniques for OSCs has led to significant progress in organic electronics, reflecting a growing understanding of material behaviors and processing methods. Various approaches to patterning OSCs, including surface-grafting polymers, capillary force lithography, wettability, evaporation assistant, and diffusion-based methods, have collectively enhanced the capability to produce high-resolution, high-performance organic electronic devices by controlling the physical and chemical energies. These techniques have demonstrated their ability to control the morphology and alignment of OSCs, improving the performance and reliability of the devices.

Surface-grafting polymers have enabled intricate patterning and enhanced functionality of organic materials. Capillary force lithography has provided a means for high-resolution patterning and single-crystal formation, contributing to the fabrication of advanced organic thin film transistors. Wettability has proven effective in controlling the deposition and organization of organic semiconductor films, leading to uniform, high-performance devices. Evaporation-assisted methods have facilitated the production of highly ordered surface patterns, while diffusion-based techniques have offered a simple and efficient approach for micro-patterning and the integration of functional molecules.

In particular, a delicate process technology capable of patterning OSCs without physical and chemical damage is essential, and efforts are needed to achieve this. More specifically, the following aspects should be referenced and developed: (1) whether OSCs can be patterned independently of the underlying substrate; (2) the feature size of each patterning technology; (3) whether the process is solvent-free or etchant-free; (4) whether large-area patterning is possible (up to wafer size limits); (5) the cost of the patterning process; and (6) whether the chemical bonds or composition of OSCs remain unchanged before and after patterning. By consistently checking these criteria and dedicating efforts to developing OSC patterning technology, it is expected that polymer-based unit electronic devices can further advance into integrated systems.

Overall, the integration and optimization of these patterning methods have significantly advanced the field of organic electronics, paving the way for developing more efficient, scalable, and versatile organic semiconductor devices. The continued development of these technologies is expected to accelerate advances in organic electronics, offering new possibilities across a wide range of applications, from flexible electronic devices to next-generation semiconductor technologies. Furthermore, the combination of advantages such as cost-effectiveness, flexibility, deformability, and light weight in organic semiconductors, along with improved crystallinity achieved through the reviewed patterning techniques, is expected to provide significant competitiveness in the organic semiconductor industry [4]. Finally, with growing environmental concerns, the introduction of biodegradable materials, such as polyvinyl alcohol, will open up opportunities for novel patterning techniques that generate less waste and pollution.

## Figures and Tables

**Table 1:** Summarization of OSC patterning techniques.

Patterning Method	Organic Material	Pattern Resolution	Device Type	Device Performance	[Ref.]
Surface-grafting polymers	p(DMAEMA)	20 μm	N/A	N/A	[54]
Surface-grafting polymers	TIPS-pentacene	46.4–105 μm	OTFT	Carrier mobility = 1.2 cm^2^·V^−1^·s^−1^	[57]
Capillary force lithography	C8-BTBT 1D single crystal	~3 μm	OFET	Carrier mobility = 5.7 cm^2^·V^−1^·s^−1^	[55]
Capillary force lithography	C8-BTBT 1D single crystal	~3 μm	OFET	Carrier mobility = 8.7 cm^2^·V^−1^·s^−1^	[60]
Wettability	C8-BTBT single crystal	100 μm	OFET	Carrier mobility = 9.3 cm^2^·V^−1^·s^−1^	[51]
Wettability	C8-BTBT/PTAA single crystal	300–400 μm	OTFT	Carrier mobility = 20.6 cm^2^·V^−1^·s^−1^	[58]
Wettability	PS nanoparticle	30 μm	N/A	N/A	[106]
	LB medium ^1^	30 μm	N/A	N/A	
	PEDOT:PSS ink ^2^	200 μm	N/A	N/A	
Evaporation Assistant	PS	4–6 μm	N/A	N/A	[116]
	PSF ^3^	4–6 μm	N/A	N/A	
	PES ^4^	4–6 μm	N/A	N/A	
Evaporation Assistant	DMQA nanowire	30–40 μm	MSM ^5^	The slope of the I–V curve = 3.1 × 10^−12^ S	[117]
Diffusion	PS	0.024 μm	N/A	N/A	[137]
Diffusion	PFO	4–25 μm	N/A	N/A	[138]

^1^ Luria–Bertani broth. ^2^ poly(3,4-ethylenedioxythiophene)-poly(styrenesulfonate) (PEDOT:PSS). ^3^ polysulfone (PSF). ^4^ polyethersulfone (PES). ^5^ metal–semiconductor–metal (MSM).

## Data Availability

Not applicable.
